# The Effects of Transcription Directions of Transgenes and the gypsy Insulators on the Transcript Levels of Transgenes in Transgenic Arabidopsis

**DOI:** 10.1038/s41598-017-15284-x

**Published:** 2017-11-07

**Authors:** Weijia Jiang, Li Sun, Xiaojie Yang, Maohua Wang, Nardana Esmaeili, Necla Pehlivan, Rongli Zhao, Hong Zhang, Yun Zhao

**Affiliations:** 1 Key Laboratory of Bio-resources and Eco-environment (Ministry of Education), College of Life Sciences, Sichuan University, Chengdu, Sichuan 610064 China; 2 Department of Biological Sciences, Texas Tech University, Lubbock, Texas 79409 USA; 3 Economic Crops Reseach Institute, Henan Academy of Agricultural Sciences, Zhengzhou, Henan, 450002 China; 4 Department of Biology, Recep Tayyip Erdogan University, 53100 Rize, Turkey; 5 Chengdu Institute of Biological Products Co., Ltd, Chengdu, Sichuan, 610000 China

## Abstract

Manipulation of a single abiotic stress-related gene could improve plant performance under abiotic stress conditions. To simultaneously increase plant tolerance to multiple stresses, it is usually required to overexpress two (or more) genes in transgenic plants. The common strategy is to assemble two or more expression cassettes, where each gene has its own promoter and terminator, within the same T-DNA. Does the arrangement of the two expression cassettes affect expression of the two transgenes? Can we use the Drosophila *gypsy* insulator sequence to increase the expression of the two transgenes? Answers to these questions would contribute to design better transformation vectors to maximize the effects of multi-gene transformation. Two Arabidopsis genes, *PP2A-C5* and *AVP1*, and the *gypsy* insulator sequence were used to construct six transformation vectors with or without the *gypsy* insulator bracketing the two expression cassettes: uni-directional transcription, divergent transcription, and convergent transcription. Total RNAs were isolated for reverse transcription- quantitative real-time polymerase chain reaction (RT-qPCR) assays and a thorough statistical analysis was conducted for the RT-qPCR data. The results showed that the *gypsy* insulator does promote the expression of two transgenes in transgenic plants. Besides, the plants containing the divergent transcription cassettes tend to have more correlated expression of both genes.

## Introduction

Since 1983, transgenic technology has been a popular method for modifying target plants to enhance their adaptation to various environmental challenges^1^. For examples, overexpression of the Arabidopsis vacuolar membrane Na^+^/H^+^ reverse transport protein 1 gene (*AtNHX1*) that encodes the vacuolar membrane bound protein/sodium antiporter leads to increased salt tolerance^2^, whereas overexpression of the transcriptional factor C-repeat/DRE Binding Factor 1 gene (*CBF1*) leads to enhanced cold tolerance^3,4^. In general, overexpressing genes encoding functional proteins, such as enzymes or channel proteins, lead to specific but limited changes in transgenic plants; overexpressing genes encoding regulatory proteins such as transcriptional factors tend to cause side effects in transgenic plants including negative impacts on plant growth and development^5,6^. Consequently, in order to obtain better results in transgenic plants, we must optimize transgene expression either by co-overexpressing multiple functional protein genes or fine-tuning the expression of regulatory genes. Thus, it is vital to develop multiple gene transformation technologies which could transfer two or more genes into plants to obtain remarkable changes in plant phenotype or to establish a new metabolic pathway in plants^7^. Studying the interaction between two co-expressing genes was the first step toward a multiple gene transformation system. Multiple genes could be stacked in transgenic plants by transforming linked genes (multiple genes on the same plasmid), crossing different transgenic plants, or performing sequential or co-transformations^8^. Among these multiple gene transformation technologies, the transformation of linked genes has been regarded as the most convenient and reliable method so far^9^.

Drought and high salinity are two major abiotic stresses threatening agricultural productivity and food security. At present, over 20% of irrigated lands are affected by drought and salt stresses in many countries^10^, and the situation is continually deteriorating. The Arabidopsis vacuolar H^+^-pyrophosphatase 1 (AVP1) is encoded by a 3,530-bp single copy gene with seven introns. The product of *AVP1* is a proton pump located on vacuolar membranes, and overexpression of *AVP1* increases drought and salt resistance in transgenic Arabidopsis^11^. Overexpression of *AVP1* establishes a higher H^+^ gradient that energizes a secondary transporter, such as Na^+^/H^+^ antiporter, to uptake more K^+^, reducing the toxicity of Na^+^, which leads to enhanced salt resistance in plants^12^. *AVP1* has been introduced into tomato^13^, tobacco^14^ and cotton^15^ to improve drought or salt resistance in these species. Moreover, the polar transport of auxin, which regulates the development of roots and sprouts, is also stimulated by overexpression of *AVP1* ^16^. Co-overexpression of *AVP1* and *AtNHX1* by crossing *AVP1*-overexpressing cotton with *AtNHX1*-overexpressing cotton has been achieved to further improve salt resistance in cotton^17^.

Protein phosphatase 2A (PP2A) is a subgroup of the very complex serine/threonine protein phosphatase family and consists of a scaffolding subunit, a regulatory subunit, and a catalytic subunit. *PP2A-C5* is one of the five genes encoding the catalytic subunit of PP2A in Arabidopsis, and it plays an important role in plant salt resistance. Overexpression of *PP2A-C5* leads to increased salt tolerance in transgenic plants and the loss of function mutant of this gene, *pp2a-c5-1*, is sensitive to salt treatment^18^. A hypothesis about how PP2A-C5 participates in plant response to salt stress suggests that PP2A might activate the chloride channel (CLC) proteins CLCa and CLCc on vacuolar membrane by removing the phosphate group from CLCa and CLCc^18^. Both CLCa and CLCc use proton as the driving force to move chloride and nitrate ion into vacuole^19^, and the proton gradient is generated by AVP1 and ATPase on the vacuolar membrane. We thought that perhaps it was possible to further increase salt tolerance by co-overexpressing *AVP1* and *PP2A-C5*, as overexpression of *AVP1* provides more protons inside vacuoles that can be used by CLCa and CLCc to exchange for chloride and nitrate, and overexpression of *PP2A-C5* activates CLCa and CLCc. To achieve this goal, we need to construct a vector that would allow us to efficiently express both *AVP1* and *PP2A-C5*.

The construction of the transformation vector is the first step in generating transgenic plants and this step plays an important role in transgene expression efficiency. When two or more open reading frames (ORFs) are integrated on one plasmid as linked genes, each exogenous gene is transcribed independently because every ORF has its own promoter, enhancer, and terminator. However, this method has a disadvantage that it is very difficult to control the transcript levels of multiple transgenes, because stacking of multiple strong promoters might cause silencing. Therefore, it will cost extra time and labor to obtain transgenic lines with the desirable level of transgene transcripts^19,20^. In addition, the transgene’s promoters might compete for RNA polymerase II, resulting in unexpected differential expression levels of two genes^21^. This could be the reason why some plants co-overexpressing multiple anti-stress genes don’t perform better than a single gene transgenic plants^22,23^. Besides, in some cases, variation of gene expression might even lead to totally unexpected plant phenotypes^24^.

The *gypsy* element, a DNA insulator, was originally found in Drosophila and is 395-bp long^25^. It blocks influences from nearby enhancers or prevents the heterochromatinization of chromosomes^26,27^. It may increase the expression of reporter genes when it is inserted at the two flanks of the reporter gene^28^. However, very few studies have been reported on the use of *gypsy* to improve transgene expression in plants. In this study, we constructed three two-gene expression vectors that had uni-directional (→→), divergent (←→), and convergent (→←) arrangements for transcription direction of transgenes *PP2A-C5* and *AVP1*, as well as three vectors that had the same arrangements for *PP2A-C5* and *AVP1* with additional *gypsy* elements on both sides of the two transgene cassettes. The transcript levels of *PP2A-C5* and *AVP1* were analyzed to investigate the role of vector’s structure and *gypsy* insulator in the transcription of both genes. Our results can be used to guide vector design for less labor intensive and more efficient multiple transgene expression.

## Results

### Construction of six two-gene co-transformation vectors and Arabidopsis transformation

**Figure 1:**
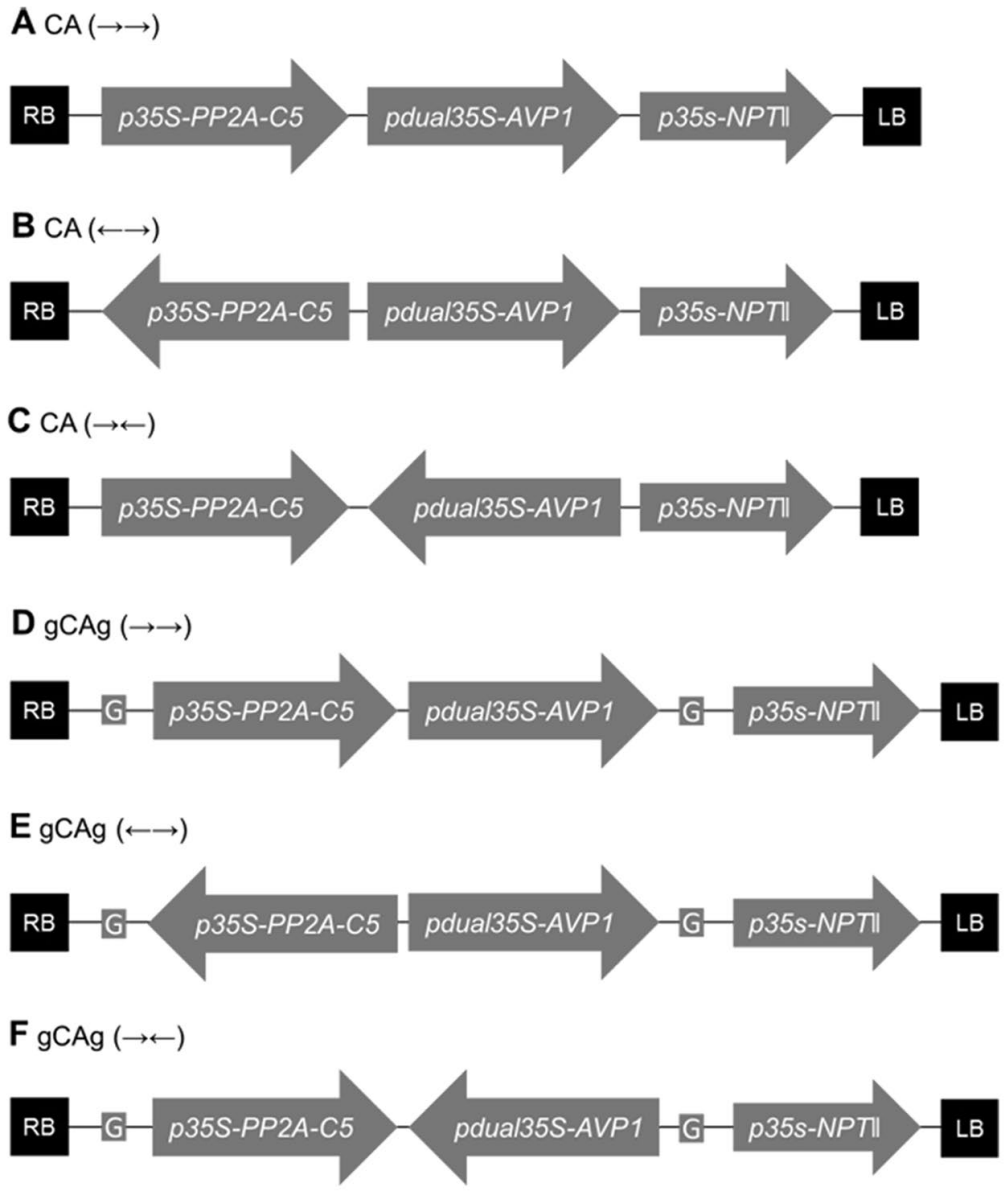
Schematic overview of six two-gene co-transformation vectors. The positions and relative orientations of *PP2A-C5* with its promoter and terminator sequences (*p35S-PP2A-C5*), *AVP1* with its promoter and terminator sequences (*pdual35S-AVP1*), and a selective marker gene with its promoter and terminator sequences (*p35S-NPTII*) are shown with respect to the right border (RB) and the left border (LB) of the T-DNA regions. A and D, uni-directional transcription vectors for *PP2A-C5* and *AVP1* expression cassettes (→→); B and E, divergent transcription vectors for *PP2A-C5* and *AVP1* expression cassettes (←→); C and F, convergent transcription vectors for *PP2A-C5* and *AVP1* expression cassettes (→←). *Gypsy* elements (G) bracket the *PP2A-C5* and *AVP1* expression cassettes in vectors D, E, and F.

To study the impact of expression cassettes arrangement and the *gypsy* insulator in the T-DNA on transgene transcription in transgenic plants, we constructed six two-gene co-overexpressing vectors for Arabidopsis transformation (Fig. 1). The two genes used are 35S promoter driven *PP2A-C5* and dual-35S promoter driven *AVP1* (see Materials and Methods). The neomycin resistance gene *NPTII* was used as the selective marker in plant transformation (Fig. 1). The two expression cassettes without or with the *gypsy* insulator bracketing transgene cassettes are arranged in the following three ways: 1) uni-directional transcription of *PP2A-C5* and *AVP1* (→→, vectors A and D), divergent transcription (←→, vectors B and E), and convergent transcription (→←, vectors D and F). No *gypsy* insulators are in the vectors A, B and C, whereas the *gypsy* insulator flanks the two expression cassettes in vectors D, E and F (Fig. 1). The directions of transgene cassettes and the presence of *gypsy* insulators were verified using polymerase chain reaction (PCR) and agarose gel electrophoresis (Supplementary Figs S1 and S2)

These six transforming vectors were introduced into wild-type Arabidopsis plants (ecotype Columbia, Col) using the ‘floral dip’ method of Clough and Bent^29^. In total, 292, 828, 289, 243, 309, and 219 independent T_1_ transgenic plants containing vectors A, B, C, D, E and F, respectively, were obtained. Then, 45 seeds of each independent transgenic line were screened on Murashige and Skoog (MS) plates supplemented with kanamycin (30 mg L^−1^) for segregation analysis. Plants that showed roughly 3 to 1 segregation ratio of kanamycin resistance to sensitivity phenotype were identified as potential single T-DNA insertion plants. 100, 70, 98, 92, 99 and 88 independent putative single T-DNA insertion transgenic lines containing vectors A, B, C, D, E and F, respectively, were obtained. These 547 transgenic plants were used for further analyses.

### Validating RT-qPCR results by RNA blot

**Figure 2:**
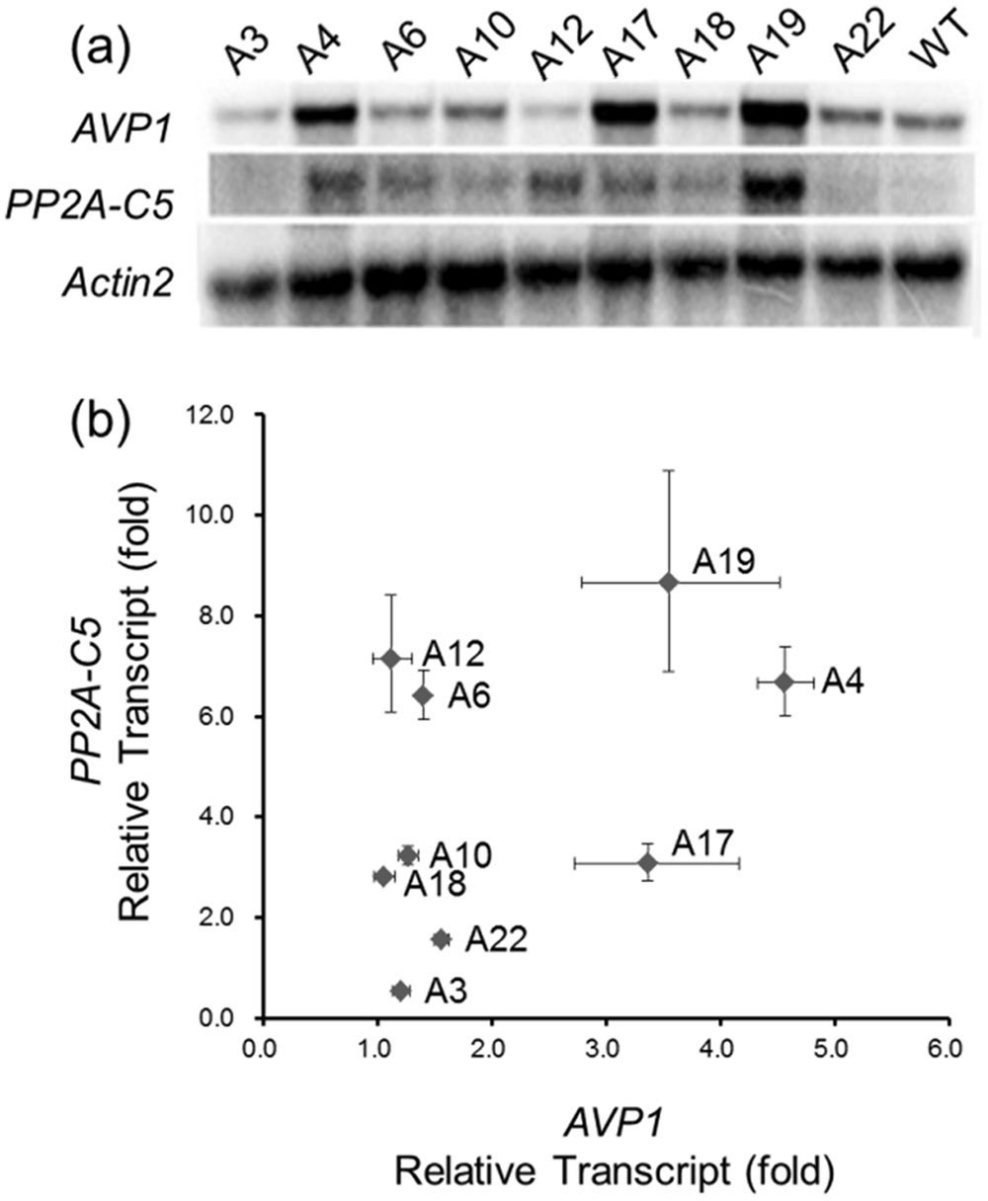
Correlation of RNA blot data and quantitative PCR data. (**a**) RNA blot analysis of nine independent *AVP1/PP2A-C5* co-expressing plants. WT, wild-type plant; A3 to A22, nine randomly selected independent *AVP1/PP2A-C5* co-expressing plants. The probes used are indicated on the left. The gene *Actin2* was used as RNA loading control. The blots are cropped and the full-length blots are presented in Supplementary Fig. S4. (**b**) Quantitative PCR analysis of the nine randomly selected *AVP1/PP2A-C5* co-expressing plants. Each dot represents an independent transgenic line. The transcript levels of *PP2A-C5* and *AVP1* relative to the level of *Actin2* in wild-type plant were set as 1. The fold of change for *AVP1* or *PP2A-C5* transcript was used to reflect the overexpression levels of transgenes in transgenic plants. The *y*-axis represents the fold of change for *PP2A-C5* transcript. The *x*-axis represents the fold of change for *AVP1* transcript. Each data point is the mean value of three repeats in RT-qPCR. Error bars represent standard deviations.

To analyze gene expression, two methods are commonly used to analyze gene transcript level: RNA blot analysis and reverse transcription-quantitative real-time polymerase chain reaction (RT-qPCR) analysis. RNA blot results are semi-quantitative and highly reliable, while RT-qPCR results are more quantitative. The RT-qPCR was used to analyze the transcript levels of *PP2A-C5* and *AVP1* in the six transgenic populations. To make sure that the RT-qPCR results are accurate and trustable, we randomly selected 9 independent transgenic lines from the transgenic plants containing vector A and analyzed the *PP2A-C5* and *AVP1* transcript levels with both RNA blot and RT-qPCR methods. The RNA blot results showed that transgenic lines A4, A17 and A19 contained high *AVP1* transcript level as well as significantly increasing *PP2A-C5* transcript level in A19. And the transcript levels of *PP2A-C5* and *AVP1* were both relatively low in lines A3 and A22 (Fig. 2a). Similar results were obtained by RT-qPCR method (Fig. 2b). This suggested that RT-qPCR could serve as a reliable tool in analyzing transcript levels of both transgenes in transgenic plants.

### All vectors can overexpress *AVP1* and *PP2A-C5*

**Figure 3:**
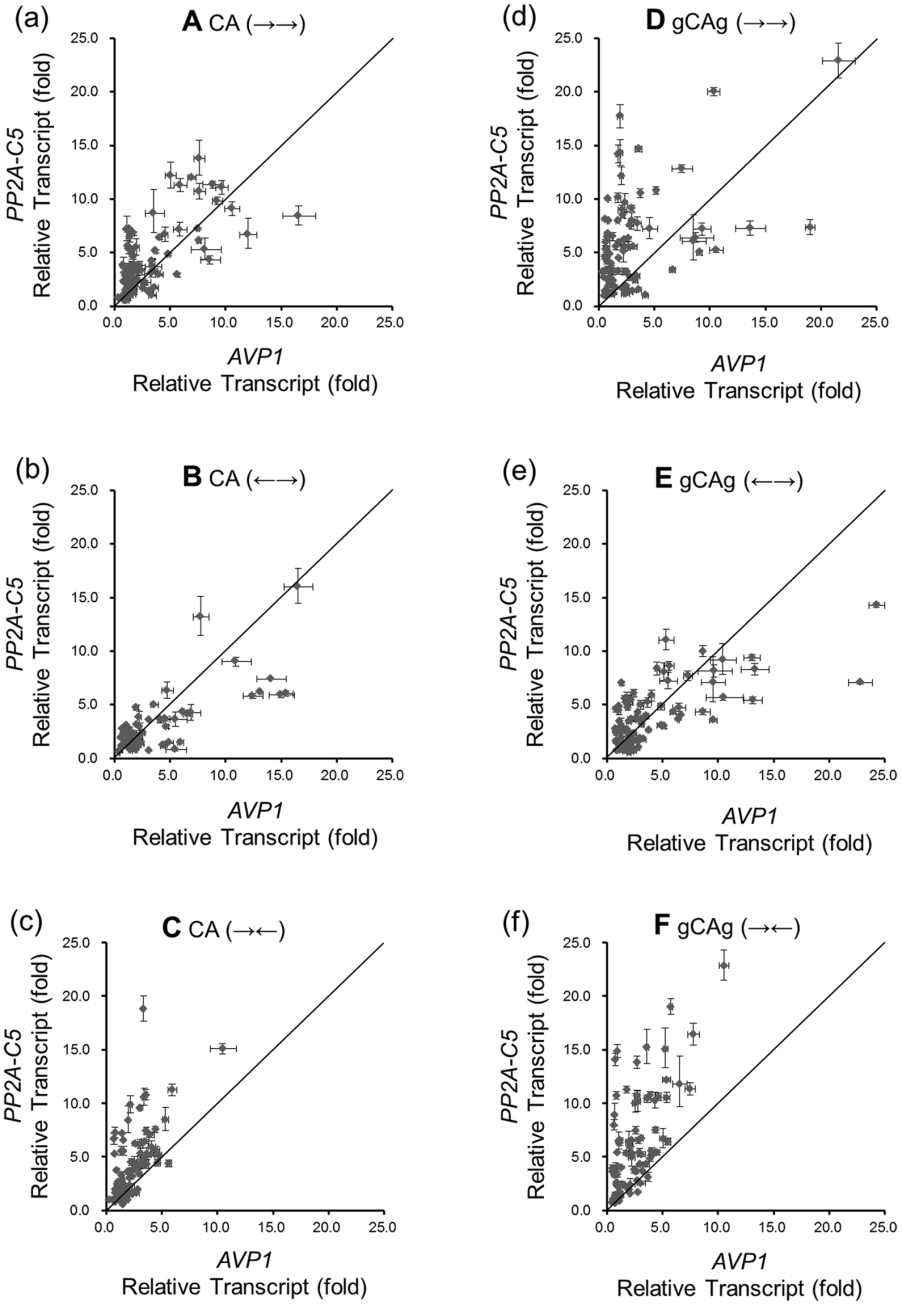
The distribution of transcript levels of *PP2A-C5* and *AVP1* in six transgenic populations. (**a**), (**b**), (**c**), (**d**), (**e**) and (**f**) Quantitative PCR data of transgenic plants containing the corresponding vector. Each dot represents a single T-DNA insertion line. The transcript levels of *PP2A-C5* and *AVP1* relative to that of *Actin2* in wild-type plant were set as 1. The fold of change for *AVP1* or *PP2A-C5* transcript was used to reflect the increased transcript levels of transgenes in the transgenic plants. The *y*-axis represents the fold of change for *PP2A-C5* transcript. The *x*-axis represents the fold of change for *AVP1* transcript. The dots near the diagonal (solid lines) represent the transgenic lines which have a ‘balanced’ *PP2A-C5* and *AVP1* overexpression level. Each data point is the mean value of three repeats of RT-qPCR analyses. Error bars represent standard deviations.

The total RNAs from all single T-DNA insertion lines were extracted and used for reverse transcription, followed by RT-qPCR analysis. The transcript levels of *AVP1* and *PP2A-C5* in wild-type plants were set as 1 and the fold of change (FOC) in *AVP1* or *PP2A-C5* transcript was used to reflect the transcription levels of transgenes in transgenic plants. A value of over 1 indicates increased transcript for transgene, a value of smaller than 1 indicates decreased transcript for the transgene being analyzed. The summary of results of 1094 RT-qPCR analyses (547 for *AVP1* and 547 for *PP2A-C5*) are shown in Table 1 and the calculated proportions of transgene overexpression lines are shown in Table 2. To better illustrate the FOC in *PP2A-C5* and *AVP1* transcript in these six transgenic populations, the FOC for *AVP1* and *PP2A-C5* in each individual plant in a particular transgenic population are shown as the Scatter diagrams in Fig. 3. It is clear that both *PP2A-C5* and *AVP1* are overexpressed in most of these transgenic plants (>72%, Table 2). The average FOC for *PP2A-C5* ranged from 2.829 to 5.977, while the average FOC for *AVP1* ranged from 2.233 to 3.783 (Table 1). Evidently, all six co-transforming vectors can be used for co-overexpressing both transgenes in Arabidopsis. Furthermore, our results also indicate that transgenic plants independently containing uni-directional or convergent transcription cassettes (vectors A, C, D and F) have higher FOC in *PP2A-C5* transcript compared with that of *AVP1* and transgenic plants independently containing divergent transcription cassettes (vectors B and E) have a more similar, or ‘balanced’ (points distributed more evenly on both sides of line y = x in Fig. 3) FOC for *AVP1* and *PP2A-C5* transcripts (Fig. 3). This interesting difference is discussed more in later sections.

**Table 1:** The increased transcript levels (in fold of change for transcript compared with that of wild-type plants) of *PP2A-C5* and *AVP1* in transgenic plants containing different vector constructs.

Gene	Group	Number of samples	Mean	Variance	Quantiles				
					Min	25%	Median	75%	Max
***PP2A-C5***	A	100	4.004	9.050	0.518	1.868	3.028	5.451	13.766
	B	70	2.829	7.425	0.540	1.195	1.888	3.606	16.011
	C	98	3.881	10.315	0.575	1.569	2.768	5.316	18.804
	D	92	5.465	18.794	0.960	2.322	4.532	7.322	22.896
	E	99	3.664	7.464	0.555	1.543	2.998	5.202	14.289
	F	88	5.977	21.660	0.678	2.395	5.107	8.666	22.848
***AVP1***	A	100	2.991	8.140	0.398	1.299	1.722	3.524	16.553
	B	70	3.639	15.085	0.439	1.346	2.069	4.595	16.507
	C	98	2.233	2.179	0.284	1.281	1.786	3.044	10.454
	D	92	2.946	13.228	0.295	0.884	1.928	2.990	21.541
	E	99	3.783	16.436	0.764	1.621	2.269	4.485	24.218
	F	88	2.459	3.634	0.431	0.964	2.020	3.428	10.498

The transcript levels of *PP2A-C5* and *AVP1* relative to that of *Actin2* in wild-type plants were set as 1. The fold of change for *AVP1* or *PP2A-C5* transcript was used to reflect the increased transcript levels of transgenes in the transgenic plants. Data collected from RT-qPCR analyses were used to calculate the mean value, variance value and quantiles of each transgenic group using IBM SPSS Statistics 22. A, B, C, D, E and F represent transgenic groups independently containing vectors A, B, C, D, E and F, respectively.

**Table 2:** The proportion of transgenic lines overexpressing the transgenes in transgenic plants containing different vector constructs.

Group	The proportion of overexpressing transgenic lines					
	A	B	C	D	E	F
*PP2A-C5* overexpression lines (relative *PP2A-C5* transcript level, folds >1)	95%	86%	92%	98%	86%	93%
*AVP1* overexpression lines (relative *AVP1* transcript level, folds >1)	92%	86%	86%	72%	93%	75%
*PP2A-C5* and *AVP1* overexpression lines (both relative *PP2A-C5* transcript level and relative *AVP1* transcript level, folds >1)	90%	76%	81%	72%	81%	74%
*PP2A-C5* and *AVP1* overexpression lines (both relative *PP2A-C5* transcript level and relative *AVP1* transcript level, folds >2)	33%	33%	39%	37%	42%	48%

The transcript levels of *PP2A-C5* and *AVP1* relative to that of *Actin2* in wild-type plant were set as 1. The fold of change for *AVP1* or *PP2A-C5* transcript was used to reflect the increased transcript levels of transgenes in the transgenic plants. The proportion of transgenic lines overexpressing *PP2A-C5* or *AVP1* in transgenic plants (upper part) and simultaneously overexpressing the two genes over 1 fold or 2 folds in transgenic plants (lower part) were shown. A, B, C, D, E and F represent transgenic groups independently containing vectors A, B, C, D, E and F, respectively.

### Transgenic plants containing vectors that include the *gypsy* insulators display higher FOCs for both transgene transcripts

To test whether the *gypsy* insulator could enhance the transcription of *AVP1* and *PP2A-C5*, the FOCs for *AVP1* and *PP2A-C5* transcripts from plants transformed with vectors D, E and F were compared with those from plants transformed with vectors A, B and C (Table 1). The *gypsy* insulators in vectors D, E and F helped transgenic plants maintain higher average FOC for *AVP1* and *PP2A-C5* transcripts (Table 1 and Fig. 3), and the variances of FOC for *AVP1* and *PP2A-C5* transcripts in transgenic plants containing the *gypsy* insulators were increased (Table 1). In addition, the *gypsy* insulators could increase the maximal value of FOC for *AVP1* transcript in populations transformed with vectors D, E or F as well as the maximal value of FOC for *PP2A-C5* in populations transformed with vectors D or F. In population transformed with the vector E, the maximal value of FOC for *AVP1* was the highest among all six transgenic populations, but the maximal value of FOC for *PP2A-C5* was lower than that of the transgenic plants transformed with vector B by contraries (Table 1). Moreover, the proportion of plants displaying higher than 2 FOC for both *AVP1* and *PP2A-C5* transcript increased in all three populations containing *gypsy* insulators (Table 2).

**Figure 4:**
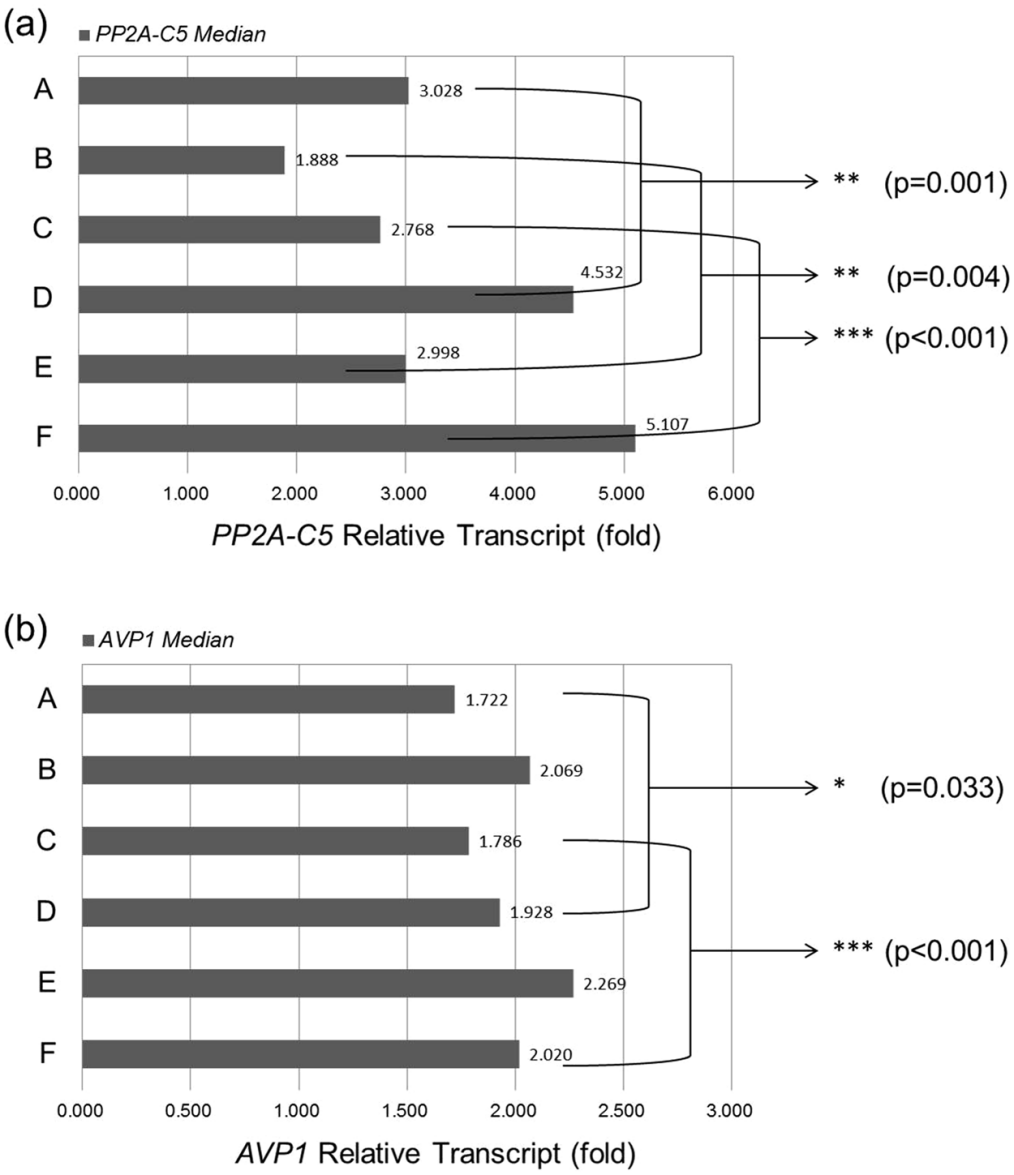
Comparison of the medians of increased transcript levels of *PP2A-C5* and *AVP1* in six groups of transgenic plants. (**a**) The medians of *PP2A-C5* transcript levels obtained from quantitative PCR analyses in six transgenic populations. (**b**) The medians of *AVP1* transcript levels obtained from quantitative PCR analyses in six transgenic populations. Because the distribution of fold of change for *PP2A-C5* or *AVP1* transcript was not within the Normal distribution (p < 0.05), the median method and Wilcoxon rank test were applied to compare these six groups of transgenic plants. The transcript levels of *PP2A-C5* and *AVP1* relative to that of *Actin2* in wild-type plant were set as 1. The fold of change for *AVP1* or *PP2A-C5* transcript was used to reflect the increased transcript levels of transgene transcripts in transgenic plants. The *x*-axis represents the fold of change for *PP2A-C5* transcript (**a**) or *AVP1* transcript (**b**). The transgenic plants containing corresponding vector were labeled on the left. An asterisk indicates a significant difference in comparisons between plants populations containing vectors A and D, B and E, and C and F, respectively. Statistical significance was determined by a Wilcoxon rank test at p < 0.05.

To further evaluate *gypsy*’s effects on gene expression, the FOC for *AVP1* and *PP2A-C5* transcripts in six groups of transgenic plants were analyzed using the software of IBM SPSS Statistics 22. The single-sample Kolmogorov–Smirnov test showed that the distributions of FOC for *AVP1* and *PP2A-C5* transcripts were not within the Normal distribution (p < 0.05). Thus, the median method and Wilcoxon rank test were applied to compare these six groups of transgenic plants (Fig. 4). The results indicated that the FOC for *PP2A-C5* transcript was significantly increased by 49.67%, 58.79% and 84.50% in plants independently containing vectors D, E or F, as compared with plants independently containing vectors A, B or C, respectively (Fig. 4a). Similarly, the FOC for *AVP1* transcript was increased by 11.96%, 9.67% and 13.10% in plants independently containing vectors D, E or F, as compared with plants independently containing vectors A, B or C, respectively (Fig. 4b). However, the increase of FOC for *AVP1* transcript was significant in plants independently containing vectors D or F, but not in plants containing vector E. Thus, the *gypsy* insulator can significantly increase *PP2A-C5* transcript in all three expression cassette arrangements, but can only significantly increase *AVP1* transcript in uni-directional and convergent arrangements of expression cassettes.

### Expression of *AVP1* and *PP2A-C5* is biased but correlated

**Figure 5:**
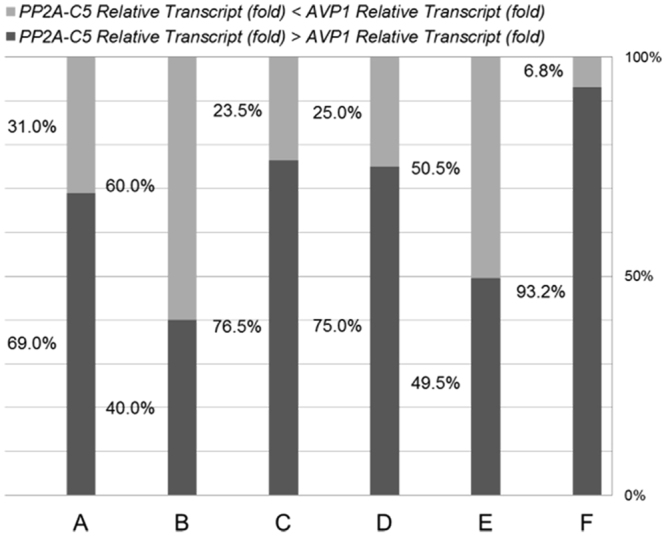
Comparisons between *PP2A-C5* and *AVP1* transcript in six transgenic populations. The transcript levels of *PP2A-C5* and *AVP1* relative to that of *Actin2* in wild-type plants were set as 1. The fold of change for *AVP1* or *PP2A-C5* transcript was used to reflect the increased transcript levels of transgenes in the transgenic plants. Data from RT-qPCR analyses were used for further calculation. Black column represents the proportion of plants in which the fold of change for *PP2A-C5* transcript was higher than that for *AVP1* transcript, and the grey column indicates the opposite situation. The transgenic populations containing corresponding vectors were labeled on the bottom.

As mentioned before, the FOC for *PP2A-C5* transcript is higher than FOC for *AVP1* transcript in four out of the six transgenic populations (Fig. 3), which might indicate different preference to express the two genes of vectors with different arrangement. The FOC for each gene’s transcript was further examined (Fig. 5). Indeed, the FOC for *AVP1* transcript was higher than that of *PP2A-C5* transcript in over 50% of the transgenic plants transformed either with vector B or vector E, while much smaller portion of plants showed higher FOC for *AVP1* transcript than that for *PP2A-C5* in plants transformed with vectors A, C, D or F (31%, 23.5%, 25.0%, and 6.8%, respectively, Fig. 5). Moreover, the divergent expression cassettes arrangement of transgenes (vectors B or E) displayed higher maximal FOC for *AVP1* transcript and lower maximal FOC for *PP2A-C5* transcript (Table 1). Considering that *AVP1* is expressed at a much higher level than that of *PP2A-C5* endogenously in Arabidopsis, this indicates a stronger bias towards expressing *AVP1* over *PP2A-C5* in plants containing the divergent expression vectors.

**Figure 6:**
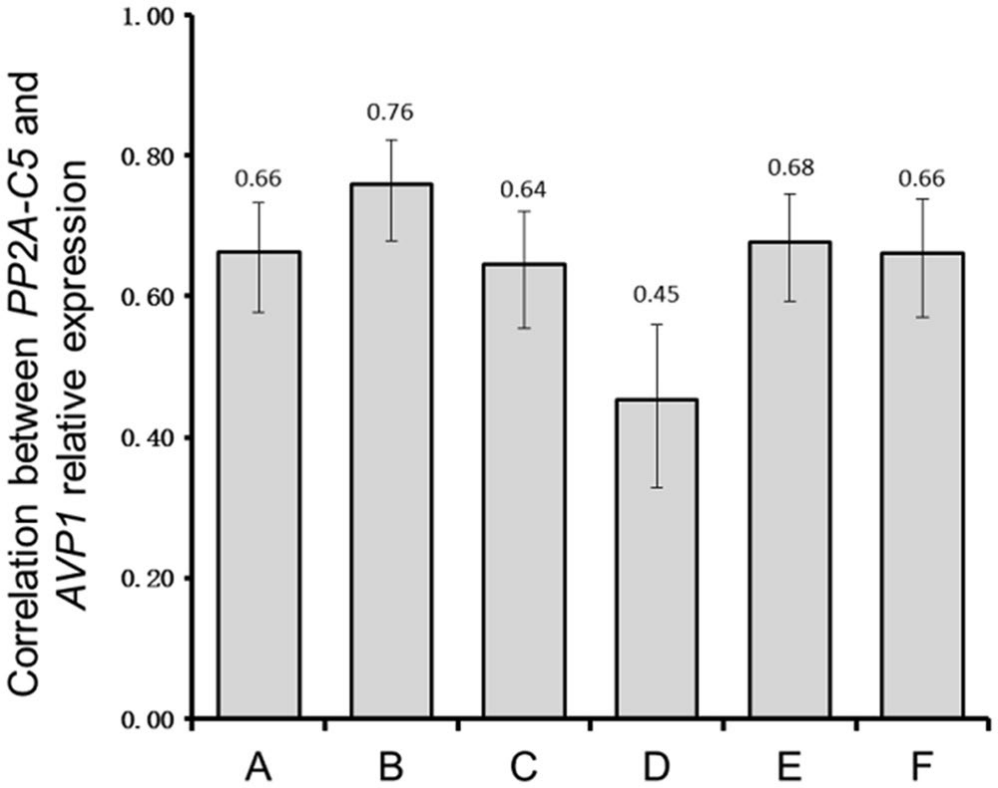
The correlation between *PP2A-C5* transcript level and *AVP1* transcript level. The transcript levels of *PP2A-C5* and *AVP1* relative to that of *Actin2* in wild-type plants were set as 1. The fold of change for *AVP1* or *PP2A-C5* transcript was used to reflect the increased transcript levels of transgenes in the transgenic plants. Data from RT-qPCR analyses were used for further calculation. The correlation value between of *PP2A-C5* transcript and *AVP1* transcript was calculated using IBM SPSS Statistics 22. The *y*-axis represents the correlation between the fold of change for *PP2A-C5* transcript and *AVP1* transcript. Values above zero represent the increased transcript levels of *PP2A-C5* and *AVP1* are positively correlated and the closer the value is to 1, the stronger the correlation between overexpression of the two transgenes is. To compare the correlation coefficients, Fisher’s Z is used to constructs the 95% confidence intervals for all groups. Error bars indicate lower and upper values of confidence intervals. A non-overlapping of confidence intervals between two data groups indicates a significant difference. The transgenic plants populations containing corresponding vector were labeled on the bottom.

To further study the expression pattern of these two transgenes in these transgenic plants, we analyzed the correlation of *AVP1* transcript’s FOC and *PP2A-C5* transcript’s FOC. There is a significant positive correlation between the up-regulated *AVP1* transcript level and *PP2A-C5* transcript level in all of these plants (Fig. 6). The highest correlation was found in plants containing vector B (0.76) and the lowest correlation was observed in plants containing vector D (0.45) (Fig. 6). Interestingly, plants containing constructs with the *gypsy* elements bracketing the two gene expressing cassettes showed lower correlations in comparison with plants containing constructs without *gypsy* elements in two out three arrangements (unidirectional and divergent) (Fig. 6). We used Fisher’s Z test to compare the correlation values among these six transgenic populations as well and found that the correlation in transgene transcriptions in plants containing vector D was significantly lower than those in plants containing vectors A, B, E or F. Moreover, the FOC for *AVP1* and *PP2A-C5* transcript appeared to be more closely related (higher correlation) when the two transgenes transcribed divergently (vectors B and E), with or without the *gypsy* element (Fig. 6).

## Discussion

We have demonstrated that co-transformation of *PP2A-C5* and *AVP1* expression cassettes arranged in three different ways, either with or without the *gypsy* insulator, can increase the transcript levels of *PP2A-C5* and *AVP1* in Arabidopsis transgenic plants. In plants populations with single T-DNA insertion, 33% to 48% of total showed higher than 2 FOC for both genes’ transcripts compared with wild-type plants (Table 2). This proved the concept that expression of linked genes is an effective way to obtain transgenic plants overexpressing two genes, consistent with the conclusion of previous investigation^8^. Previous study reported that while introducing exogenous genes into plants, there are three major factors that could affect the expression of transgenes: copy number of transgene, location of integration, and complex mechanisms of regulation^30–32^. In our study, in order to remove influences of multiple copies of transgenes and the positional effects of T-DNA insertions, 100, 70, 98, 92, 99, and 88 independent single T-DNA insertion lines were analyzed. Although the latter two factors could not be controlled easily, it is practicable to alleviate their impact by increasing the number of independent transgenic lines for each transformation vectors. Analysis of relatively large population can statistically eliminate the influence of rare events caused by unknown mechanisms. Additionally, because we used the FOC for *AVP1* and *PP2A-C5* transcript in transgenic plants compared with wild-type, which is a relative quantitative method, to reflect the transcription levels of transgenes, it also remove the background deviation caused by transgenic technology itself. Also, to increase the accuracy of our detection, RT-qPCR was used to evaluate the FOC for gene’s transcript and confirmed with RNA blot. Thus, we believed that our study could correctly reveal the influence of transgene expression cassettes arrangement and the *gypsy* insulator on the transcription of *PP2A-C5* and *AVP1* in transgenic Arabidopsis.

The main purpose of our study was to investigate the effects of different expression cassette arrangements on transgene expression. Plant characteristics can be dramatically changed by simply increasing the expression of a particular transcription factor gene^33^. However, overexpressing an exogenous transcription factor gene could result in negative effects on plant growth^6^ and different expression patterns of transgenes in multi-gene expression cassettes may also lead to totally opposite phenotypes^34,35^. Moreover, co-overexpression of two or more genes is an energy consuming process and can place a large burden on plant growth and development. For example, when a whole metabolic pathway in plants needs to be modified or established, how to control the precise expression of each transgene in multi-gene expression cassettes is extremely important. Thus, the assembly of co-expressing genes and elements plays an important role in the construction of multi-gene expression cassettes. Here, we assembled *PP2A-C5* and *AVP1* expression cassettes in three different ways, and all three arrangements could increase the transcription of both transgenes. The results suggested that if *PP2A-C5* and *AVP1* expression cassettes were assembled in a divergent way, the changes of their transcription level were similar to each other and more correlated to each other (Table 1, Figs 3 and 6). We assume that when *PP2A-C5* and *AVP1* were transcribed divergently (Fig. 1), a bi-directional promoter structure was created^36^ and the RNA polymerase II could bind to the promoter of both expression cassettes in a coordinated fashion. On the contrary, in plants independently containing vectors A, C, D or F, in which the *PP2A-C5* and *AVP1* expression cassettes were either assembled in uni-directional or convergent way, the transcription of *PP2A-C5* was enhanced and correlation between FOC of *AVP1* and *PP2A-C5* was decreased compared with those in plants containing vectors B or E (Table 1, Figs. 3 and 6). This might be due to several factors. Firstly, distance between the two promoters might lead to more independent transcription initiation and less correlation between their transcriptions. Secondly, readthrough of RNA polymerase II might affect transcription of downstream genes, such as *AVP1* and *PP2A-C5* expression cassettes in unidirectional or convergent arrangements. This could be due to antisense-RNA or even simply steric hindrance suppressing RNA Polymerase II assembly. In our case, *PP2A-C5* is much shorter than *AVP1*, which might cause more readthrough and stronger negative effect on *AVP1*. The similar transcriptional suppression of convergently or uni-directionally transcribed genes was also noticed by Chen *et al*.^37^. Thus, we conclude that expressing two genes in divergent way can achieve a more correlated transcription of both transgenes, while expressing two genes in uni-directional or convergent way could incur more competitive and less correlated transcriptions of transgenes.

The other purpose of our study was to investigate the function of the *gypsy* insulator in the expression of two transgenes. There are applications of *gypsy* as a *cis*-element in several species^38–40^. In Arabidopsis, *gypsy* increased the expression of a reporter gene^28^. *Gypsy* not only enhanced the expression of genes located between the two *gypsy* sequences, but also enhanced the expression of genes near the *gypsy* element^28^. Blocking enhancers nearby or preventing DNA from heterochromatinization may be the two major functions of *gypsy* ^26,27^, and the functions are distance related and orientation independent^26^. In our study, we extended the investigation of *gypsy* to abiotic stress-related genes. With the addition of the *gypsy* insulator, the *PP2A-C5* and *AVP1* transcript levels tend to increase, with the former more than the latter (Table 1, Fig. 4), and the proportion of plants showing FOC for both genes’ transcripts higher than 2 folds of wild-type levels increases, too (Table 2). In the plants containing vectors D, E or F, the FOC for *PP2A-C5*’s transcript was significantly increased by 49.67%, 58.79% and 84.50%, respectively (Fig. 4a) in comparison with plants containing the similar vectors except without *gypsy*. Although the increase in *AVP1* transcription (9.67%) in plants containing vector E, compared with plants containing vector B, was not significant, the increase in plants containing vectors D or F (11.96% and 13.10%, respectively) (Fig. 4b), compared with plants containing vectors A or C, was significant. This may be due to the increase of *AVP1* transcript level in plants containing vector E was too small to be detected. Besides, in wild-type plants, the endogenous transcript of *AVP1* was 2^2^ to 2^3^ times higher than the transcript of *PP2A-C5*. This might lead to significantly higher increase in *PP2A-C5* transcript in the presence of *gypsy*. In addition, *gypsy* could also increase the variance of FOC for *PP2A-C5* and *AVP1* transcript and the maximal FOC values for *AVP1* transcript in transgenic populations containing vectors D, E, or F. Meanwhile, it could increase the maximal FOC values for *PP2A-C5* transcript in transgenic plants containing vectors D or F (Table 1). Besides, with the addition of *gypsy*, the correlation between FOC for *PP2A-C5* and *AVP1* transcripts was significantly decreased in transgenic population containing uni-directional construct (Fig. 6). The *gypsy*’s influence on correlation values in populations independently containing vectors D, E or F, compared with plants independently containing vectors A, B or C, was not significant. We assume that the *gypsy* may stabilize transcription by reducing the interference between two co-expressing genes, and the relative stronger interference between two genes in uni-directionally and convergently transcribed vectors than in divergently transcribed vector may contribute to the more significant change for genes’ transcripts in vectors D and F. Thus, we conclude that in the presence of *gypsy* insulator, the transcript level of both transgenes can be increased in all two-gene expression cassettes arrangements at the cost of increased variation and potentially decreased correlation.

Hence, our results can assist the selection of proper vector construction when designing co-expression of multiple genes for different purposes. Without adding the *gypsy* insulator, uni-directionally, divergently or convergently transcribed vector can all provide highly correlated enhanced transcription of two transgenes (Table 1, Fig. 6). The uni-directionally and convergently arranged vectors can be used to specifically overexpress one major gene and co-overexpress the other by tuning the order of the two transgenes. The divergently arranged vector can overexpress two genes in a more correlated way (Figs 3 and 5), which means less number of plants to screen for transgenic lines highly overexpressing both genes. On the other hand, with the addition of the *gypsy* insulator, uni-directionally and convergently transcribed vectors can generate transgenic plants with higher transcript level of transgenes (Table 1). Unfortunately, this increase is accompanied by increased variance within the population and reduced correlation between the enhancements of two transgenes’ transcripts, which means in order to obtain higher transcription more independent transgenic lines are needed. If it is expected to simultaneously overexpress two transgenes as highly as possible and it is easy to obtain a large number of transgenic plants, for example, in Arabidopsis, the uni-directionally and convergently transcribed vectors containing *gypsy* would be the best choice. However, in some species, such as cotton, to obtain a large number of transgenic plants to screen for high overexpression plants are time- and labor-consuming. It is better to select divergently transcribed vector containing *gypsy* for transformation due to the higher correlation. In summary, our work provides a new perspective on multiple genes overexpression in plants. This study can serve as a guideline for vector construction in biotechnology research.

## Materials and Methods

### Plant materials and growth conditions

The Arabidopsis ecotype Columbia-0 (Col-0) was used in this study. Arabidopsis seeds (approximate 20 μL) were sterilized in 15% bleach (KIK international Inc., Houston, TX, USA) for 30 min, followed by washing with sterile distilled water three times to remove the bleach. Seeds were kept in at 4°C for 24 h. Then, were suspended in 3 drops of 0.07% agarose (AMRESCO) and plated on 1/2 Murashige and Skoog (MS) plates^41^. Plants were grown under continuous light conditions at 22°C (ambient temperature). After 12 days of culturing on plates, the plants were transferred to soil and grown in a growth chamber (ENCONAIRAC-60, Ecological Chamber Inc., Winnipeg, Manitoba, Canada) with a 16 h light: 8 h dark photoperiod at a light intensity of 120 mmol s^−1^m^−2^ and 22 °C with 50% relative humidity. Distilled water was used for watering in our experiments.

### Construction of basic vector containing *gypsy*

The binary vector pPZP212, *Escherichia coli* DH5α and Agrobacterium strain GV3101 from our laboratory were used in this experiment. The *gypsy* fragments were cloned from pGYPSY-TL-Su(Hw), which was donated by Dr. Junhui Wang^28^, by PCR (Q5^®^ High-Fidelity DNA Polymerase, New England Biolabs, Beverly, MA, USA). The gypHX35 (primers: Gyp3 and Gyp-xho-hin5) and gypES35 (primers: Gyp3 and Gyp-sac-eco5) fragments were amplified. pPZP212 was digested with *Hin*dIII, treated with T4 DNA polymerase and then digested with *Sal*I to generate a linear background vector with one blunt end and one sticky end. The linear fragment was treated with alkaline phosphatase (New England Biolabs) to prevent vector’s self-ligation. Then, the *Xho*I-digested gypHX35 fragment was ligated to the linear fragment to form pPZP212-5G. pPZP212-5G was digested with *Eco*RI, treated with T4 DNA polymerase and then digested with *Sac*I after alcohol precipitation and purification to generate a new linear fragment with one blunt end and one sticky end. The *Sac*I-digested gypES35 fragment was ligated to the new fragment to form the circular plasmid pPZP212G with two *gypsy* insulators flanking the multi-cloning site. The plasmid was then ready for the insertion of the gene expression cassette.

### Construction of *PP2A-C5* and *AVP1* co-expression vectors with *gypsy*

The *PP2A-C*5 expression cassette originated from the pFGC5941-C5 plasmid^18^. The pFGC5941-C5 plasmid was partially double-digested with *Hin*dIII and *Eco*RI to release a 3,198-bp DNA fragment. This fragment contained the *PP2A-C5*, 35S promoter and the octopine synthase terminator sequence. pPZP212G was also double-digested with *Hin*dIII and *Eco*RI, and ligated with the 3,198-bp fragment to form the p212G-C5 vector. The *AVP1* gene was amplified by PCR from an Arabidopsis cDNA library using Q5^®^ High-Fidelity DNA polymerase with primers AVP1-F1 and AVP1-R1. The blunt-end *AVP1* was then ligated to pRT103 and digested with *Sma*I to form pRT103-AVP, which contained a dual 35S promoter and polyadenylation signal of the CaMV strain Cabb B-D^42^. pRT103-AVP was sequenced (Invitrogen Corp., Carlsbad, CA, USA) and then digested with *Hin*dIII to release the *AVP1* expression cassette. Meanwhile, pPZP212 and p212G-C5 were treated with *Hin*dIII and dephosphorylated by alkaline phosphatase to prevent self-ligation. These two linear fragments were ligated into the *AVP1* expression cassette to form pPZP212-AVP and two co-expression vectors, p212G-C5-AVPU (→→, named ‘vector D’) and p212G-C5-AVPC (→←, named ‘vector F’). The directions of the insertions were determined by PCR. p212G-C5-AVPU was treated with *Eco*RI to release the *PP2A-C5* expression cassette and dephosphorylated by alkaline phosphatase. pFGC5941-C5 was treated with *Hin*dIII and alkaline phosphatase to prevent self-ligation. A 340-bp fragment was released by *Hin*dIII from pJG4-5^43^ (Clontech Laboratories, Inc., CA, USA) and then ligated with linearized pFGC5941-C5 to form pFGC5941-EC5E. Then, the *PP2A-C5* expression cassette released from pFGC5941-EC5E by *Eco*RI was ligated with linearized p212G-C5-AVPU to form p212G-C5-AVPD (←→, named ‘vector E’).

### Construction of *PP2A-C5* and *AVP1* co-expression vectors not containing *gypsy*

The *PP2A-C5* expression cassette released from pFGC5941-EC5E by *Eco*RI was ligated with linearized pPZP212-AVP after treatment with *Eco*RI and alkaline phosphatase to form p212-C5-AVPU (→→, named ‘vector A’) and p212-C5-AVPD (←→, named ‘vector B’). The *AVP1* expression cassette released from pRT102-AVP after treatment with *Hin*dIII was ligated with p212-C5-AVPU (→→) to form p212-C5-AVPC (→←, named ‘vector C’).

### Transformation of Arabidopsis

Vectors A, B, C, D, E and F were transferred into Agrobacterium and then transformed into Arabidopsis (T_0_ generation) using the ‘floral dip’ method^29^. Seeds of the T_0_ generation were surface sterilized with 15% bleach and plated on 1/2 MS medium containing 50 mg L^−1^ cefotaxime and 50 mg L^−1^ kanamycin to screen for positive transgenic plants (T_1_ generation). The putative positive transgenic plants at the four true leaf stage were transferred into soil, and the seeds (T_2_ generation) were harvested. The T_2_ generation were cultured on 1/2 MS medium containing 30 mg L^−1^ kanamycin (45 seeds per transgenic line) to select single-insertion transgenic plants using Mendel 3:1 segregation analysis. The DNA of T_2_ seedling were extracted^44^ and subjected to PCR with GoTaq DNA Polymerase (Promega, Madison, WI, USA) to identify the insertion of *gypsy*, *PP2A-C5* and *AVP1*.

### Analyses of *PP2A-C5* and *AVP1* relative expression levels

Seeds of the putative single insertion T_1_ generation were grown on 1/2 MS medium for nine days. RNA was extracted from over 50 seedlings of each line using TRIzol reagent (Invitrogen). The purity and concentration of RNA were determined (NanoDrop ND-1000, Thermo Scientific, Wilmington, DE, USA). Then 1 μg of total RNA was used for reverse transcription to make cDNA with an iScript cDNA synthesis Kit (Bio-Rad, Hercules, CA, USA). Relative quantification (RT-qPCR) analyses were carried out using SsoFast EvaGreen Supermix (Bio-Rad) on an ABI7500 (Applied Biosystems, Foster City, CA, USA) with primer pairs AVP1-F and AVP1-R, PP2A-C5-F and PP2A-C5-R, and ACTIN-F and ACTIN-R (Supplementary Table S1). Randomly selected transgenic plants transformed with vector A were subjected to northern blotting^44^ (primer pairs AVP1-F1 and AVP1-R1, C5fullF and C5FullR, and ACT2fullF and ACT2FullR; see Supplementary Table S1) to verify the RT-qPCR results.

### Statistical analyses

The relative transcript levels of *PP2A-C5* and *AVP1* in six groups of transgenic plants were analyzed using IBM SPSS Statistics 22. The mean values, medians and quantiles were compared and Wilcoxon rank test was used to analyze the significant differences at the significance level p<0.05. The single-sample Kolmogorov–Smirnov test was used to determine the data’s consistency with a Normal distribution. Fisher’s Z is used to constructs the 95% confidence intervals to compare the correlation coefficients.

## Electronic supplementary material

Supplementary Materials
