# Supplementary material for: The Effects of Transcription Directions of Transgenes and the gypsy Insulators on the Transcript Levels of Transgenes in Transgenic Arabidopsis
Source: Sci Rep. 2017 Nov 7;7:14757. doi: 10.1038/s41598-017-15284-x (PMC5676714; doi:10.1038/s41598-017-15284-x)
Supplement: Supplementary file 1 — Supplementary Materials [file 41598_2017_15284_MOESM1_ESM.pdf]

The Effects of Transcription Directions of Transgenes and the *gypsy* Insulators on the Transcript Levels of Transgenes in Transgenic Arabidopsis

Weijia Jiang[1, 6], Li Sun[2, 6], Xiaojie Yang[3, 6], Maohua Wang[1], Nardana Esmaeili[2], Necla Pehlivan[4], Rongli Zhao[5], Hong Zhang[2] and Yun Zhao[1, *]

Supplementary Materials

Identification of T_1 transgenic plants and confirmation of the transcriptional direction of the *AVP1*/*PP2A-C5* expression cassettes.

Seeds of T_1 transgenic plants were plated on MS medium. DNAs were extracted after 7 days of growth. One primer located within the vector and the other primer located within the target gene were used for PCR to confirm the orientations of the gene expression cassettes. The PCR products were used for agarose gel electrophoresis (Figure S1 and S2).

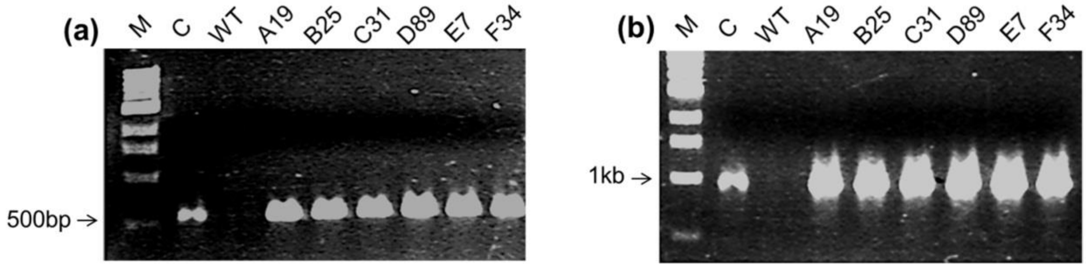

Supplementary Figure S1. Confirmation of *AVP1* and *PP2A-C5* insertion in transgenic plants. **(a)** Confirmation of *AVP1* insertion in transgenic plants. **(b)** Confirmation of *PP2A-C5* insertion in transgenic plants. M, DNA molecular weight marker (DL10000); C, plasmid positive control; WT, wild-type; A19, B25, C31, D89, E7, and F34, randomly selected DNAs from transgenic plants containing the corresponding vector construct.

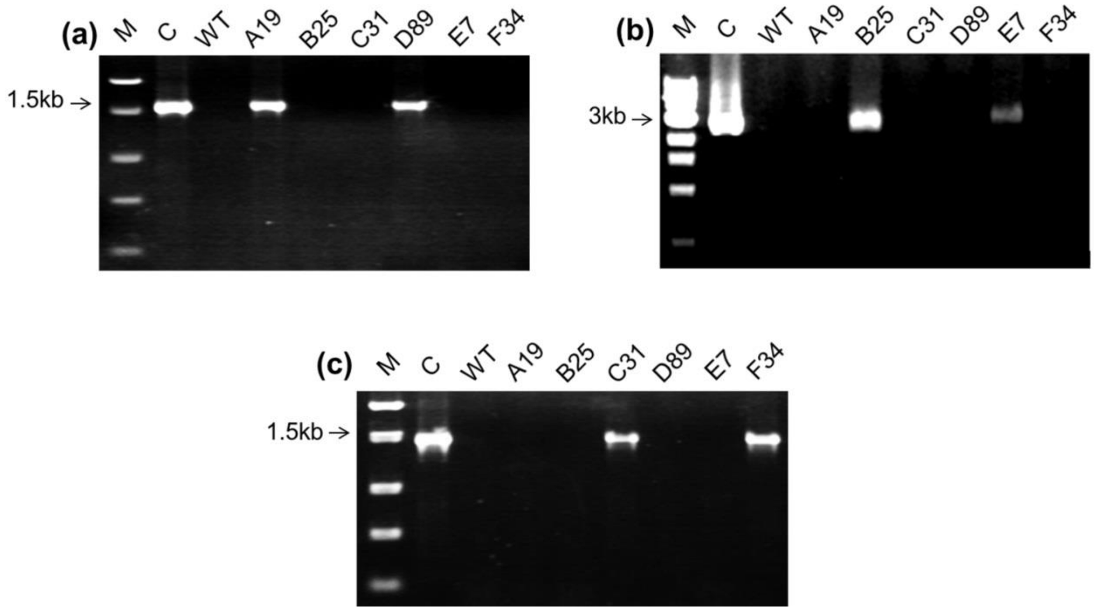

Supplementary Figure S2. Confirmation of *AVP1* and *PP2A-C5* expression cassettes arrangement in transgenic plants. **(a)** Confirmation of vector A- and vector D-containing transgenic plants. **(b)** Confirmation of vector B- and vector E-containing transgenic plants. **(c)** Confirmation in vector C- and vector F-containing transgenic plants. M, DNA molecular weight marker (DL2000); C, plasmid of vector C; WT, wild-type. A19, B25, C31, D89, E7, and F34 randomly selected DNAs from transgenic plants containing the corresponding vector constructs.

Confirmation of the insertion of the *gypsy* element in the vectors

Primers located inside *gypsy* and *PP2A-C5* were used to confirm the insertion of *gypsy* near *PP2A-C5*. And primers located inside *gypsy* and *AVP1* were used to confirm the insertion of *gypsy* near *AVP1*.

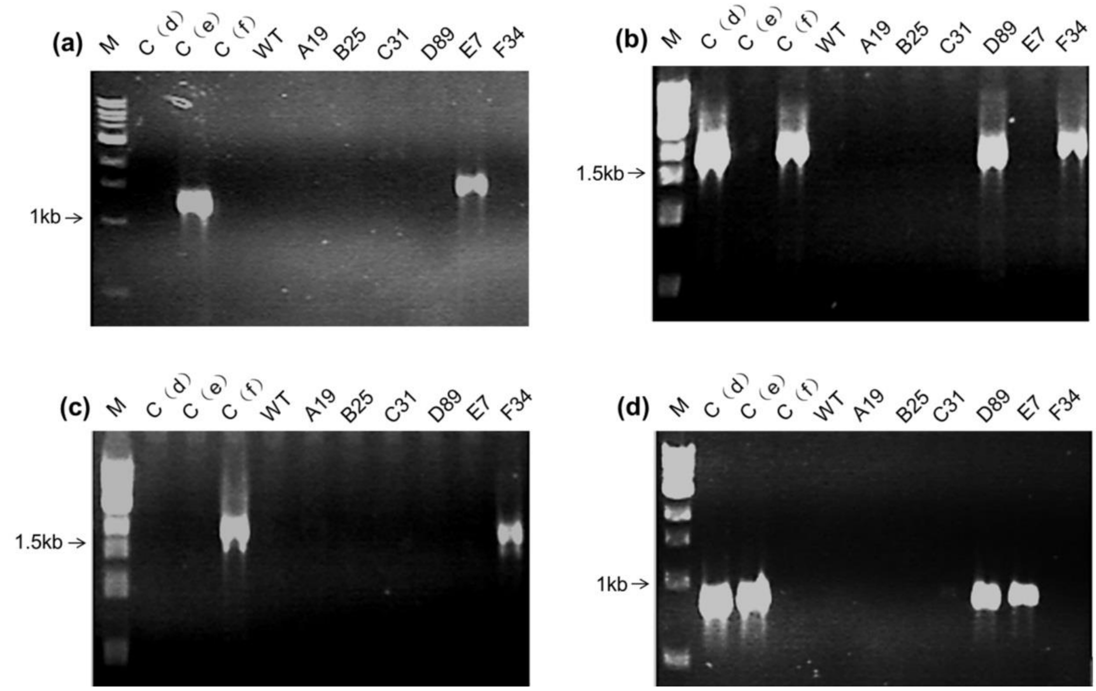

Supplementary Figure S3. Confirmation of the *gypsy* element insertion in transgenic plants. **(a)** Confirmation of *gypsy* insertion near *PP2A-C5* in vector E. **(b)** Confirmation of *gypsy* insertion near *PP2A-C5* in vector D and F. **(c)** Confirmation of *gypsy* insertion near *AVP1* in vector F. **(d)** Confirmation of *gypsy* insertion near *PP2A-C5* for in D and E. M, DNA molecular weight marker (DL10000); C (d), plasmid control of vector D; C (e), plasmid control of vector E; C (f), plasmid control of vector F; WT, wild-type. A19, B25, C31, D89, E7, and F34, randomly selected DNAs from transgenic plants containing the corresponding vector construct.

The full-length blot of RNA

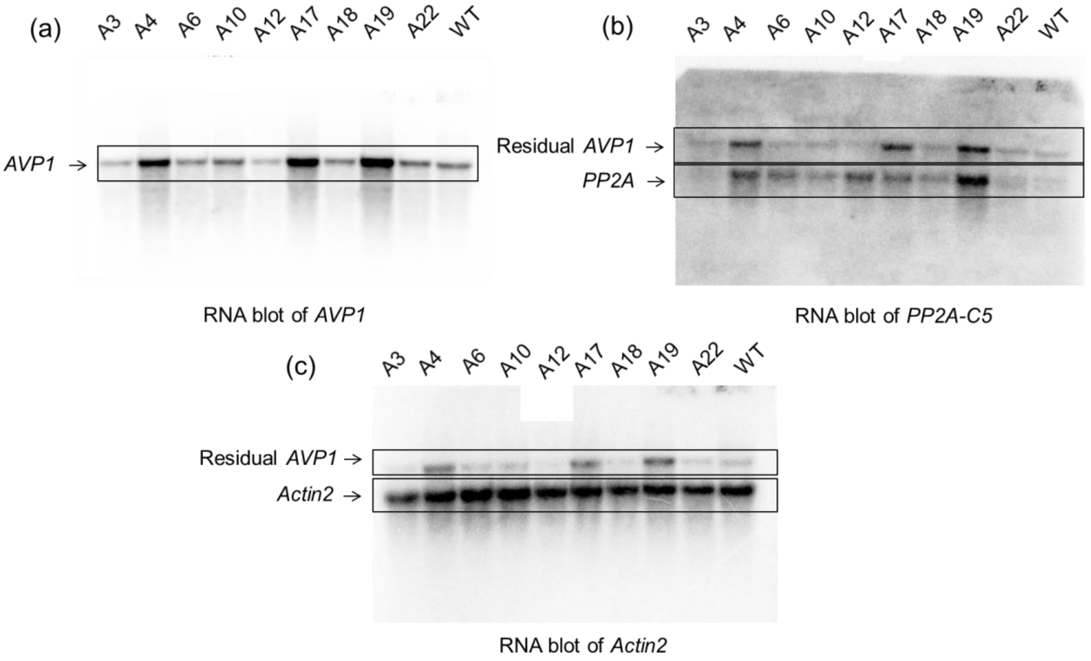

Supplementary Figure S4. The full-length blot of RNA. The samples derive from the same experiment and that three gels were processed in parallel. WT, wild-type plant; A3 to A22, nine randomly selected independent *AVP1/PP2A-C5* co-overexpressing plants. The odor to hybridize is that the first probe is *AVP1* probe, the second is *PP2A-C5* probe and the final is *Actin2* probe. **(a)** The full-length blot of *AVP1*. **(b)** The full-length blot of *PP2A*. **(c)**

The full-length blot of *Actin2*. To protect the integrity of total RNA, the strong *AVP1* is not washed thoroughly. The residual *AVP1* appears in the *PP2A-C5* and *Actin2* blots.

Supplementary Table S1 Primers

Clone of *gypsy* gypHX35

Name	Sequences
Gyp3	5'-AATTGATCGGCTAAATGGTATGG-3'
Gyp-xho-hin5	5'-AGTCCTCGAGAAGCTTTCACGTAATAAGTGTGCGTTGAATT-3'

Clone of *gypsy* gypES35

Name	Sequences
Gyp3	5'-AATTGATCGGCTAAATGGTATGG-3'
Gyp-sac-eco5	5'-AGTCGAGCTCGAATTCTCACGTAATAAGTGTGCGTTGAATT-3'

Clone of *AVP1*

Name	Sequences
AVP1-F1	5'-ATGGTGGCGCCTGCTTTGT-3'
AVP1-R1	5'-TTAGAAGTACTTGAAAAGGATAC-3'

Verification of *PP2A-C5*, *AVP1* and *Gypsy* insertion

1) *PP2A-C5* insertion

Name	Sequences
35S	5'-CCCACGAGGAGCATCGTGGAAAAAGAAGACGT-3'
C5- RTR1	5'-TTGTCCGAAAGTGTAGCCTG-3'

2) *AVP1* insertion

Name	Sequences
AVP-1857-F1	5'-CCCTGGACTTATGGAAGGAACC-3'
AVP-3UTR-R2	5'-CCTTATCTGGGAACTACTCACACATTA-3'

3) *Gypsy* close to *PP2A-C5* insertion

Name	Sequences
C5-RTF1	5’-TTAGATCGAATTCAAGAGGTTCCA-3’
C5-RTR1	5’-TTGTCCGAAAGTGTAGCCTG-3’
Gyp to 5’	5’-AAATTATTTGGTTTCTCTAAAAAGTATGC-3’

4) *Gypsy* close to *AVP1* insertion

Name	Sequences
AVP-RTR2	5’-CCGTGAATAGGAATGAAGTTGC-3’
AVP-1857-F1	5’-CCCTGGACTTATGGAAGGAACC-3’
Gyp to 5’	5’-AAATTATTTGGTTTCTCTAAAAAGTATGC-3’

5) Confirmation of Uni-directional transcription

Name	Sequences
AVP-RTR2	5’-CCGTGAATAGGAATGAAGTTGC-3’
C5-RTF1	5’-TTAGATCGAATTCAAGAGGTTCCA-3’

6) Confirmation of divergent transcription

Name	Sequences
AVP-RTR2	5’-CCGTGAATAGGAATGAAGTTGC-3’
C5-RTR1	5’-TTGTCCGAAAGTGTAGCCTG-3’

7) Confirmation of convergent transcription

Name	Sequences
AVP-RTF1	5’-TCATGCTCACACCTCTCATTG-3’
C5-RTF1	5’-TTAGATCGAATTCAAGAGGTTCCA-3’

Northern blot

Name	Sequences

AVP1-F1	5’-ATGGTGGCGCCTGCTTTGT-3’
AVP1-R1	5’-TTAGAAGTACTTGAAAAGGATAC-3’
C5FullF	5’-ATGTACCCATACGATGTTCCAGATTAC-3’
C5FullR	5’-TTACAAAAAATAATCTGGAGTCTTGC-3’
ACT2FullF	5’-ATGGCTGAGGCTGATGATATTC-3’
ACT2FullR	5’-TTAGAAACATTTTCTGTGAACGATTC-3’

Quantitative PCR

Name	Sequences
AVP1-F	5’-GTGGGATCTACACTAAGGCTG-3’
AVP1-R	5’-TCCCATACCAGCAATGTCAC-3’
PP2A-C5-F	5’-TACAGCTCTTATTGAGAGTCAG-3’
PP2A-C5-R	5’-ATGTGGAACCTCTTGAATTCG-3’
ACTIN-F	5’-CAGCATGAAGATTAAGGTCGTTG-3’
ACTIN-R	5’-TTCTGTGAACGATTCCTGGAC-3’
